# Access of high technology medicines in Russia

**DOI:** 10.1186/2052-3211-8-S1-P19

**Published:** 2015-10-05

**Authors:** Liudmila Bezmelnitsyna, Dmitry Meshkov, Ramil Khabriev

**Affiliations:** 1 National Research Institution for Public Health, Moscow, 105064, Russia

## Background

The access of high-technology medicines in Russia is restricted for such reasons as pricing and reimbursement policy, medical indications and many others. Most difficulties are presented in children because of a lack of clinical data about effectiveness and safety.

The objective of the study was to evaluate acceptability, affordability and availability of high technology medicines and provide the practical recommendations for optimization of their access in Russia (e.g.,the biologics in children with juvenile idiopathic arthritis (JIA)).

## Methods

A retrospective non-interventional study of JIA patients (405 patients in 12 federal specialized units, 6-month treatment affect by biologics and disease-modifying therapy; 2011 - 2012) for assessment of clinical and economical effectiveness of biologics and disease-modifying therapy[1]. Policy evaluation according to data from an expert's survey (63 heads and clinical specialists) from those specialized units (2013). Publically available information includes analyses of statistics, clinical recommendations and legislation (2011-2013).

## Results

Availability: six biologics (Infliximab, Adalimumab, Abatacept, Rituximab, Etanercept, Tocilizumab) were available for patients with JIA in Russia. Two of them (Infliximab and Rituximab) were used off-label. Affordability: direct medical costs for therapy including biologics were nine times higher than for disease-modifying drugs[2]. Price negotiation and risk-sharing were not used for reducing price. In 2010 only rituximab was included in the List of Vital and Essential Drugs, which means it could be added to the Federal and Regional programs of reimbursement. Others were administered only in very severe cases in Federal Research Institutions. Several national health economic research studies for biologics were held in Russia in 2010-2013. Their results helped to include the remaining biologics in the List and increase their affordability.

Acceptability: expert survey has shown that there is a very long patient journey for administration of biologics for advanced forms of JIA in patients with proved disability. There is a restriction of access to biologics on a regional level.

## Conclusions

There is an improvement of affordability of high-technology medicines in Russia thanks to development of the methods of health technology assessment.
